# Biallelic variants in *TTC21B* as a rare cause of early‐onset arterial hypertension and tubuloglomerular kidney disease

**DOI:** 10.1002/ajmg.c.31964

**Published:** 2022-03-15

**Authors:** Eric Olinger, Pran Phakdeekitcharoen, Yasar Caliskan, Sarah Orr, Holly Mabillard, Charles Pickles, Yincent Tse, Katrina Wood, Genomics England Research Consortium, John A. Sayer

**Affiliations:** ^1^ Translational and Clinical Research Institute, Faculty of Medical Sciences Newcastle University Newcastle upon Tyne UK; ^2^ Division of Nephrology Saint Louis University Center for Abdominal Transplantation St. Louis Missouri USA; ^3^ Paediatric Nephrology, Great North Children's Hospital Newcastle upon Tyne Hospitals NHS Foundation Trust Newcastle upon Tyne UK; ^4^ Department of Cellular Pathology Newcastle upon Tyne Hospitals NHS Foundation Trust Newcastle upon Tyne UK; ^5^ Renal Services Newcastle upon Tyne Hospitals NHS Foundation Trust Newcastle upon Tyne UK; ^6^ NIHR Newcastle Biomedical Research Centre Newcastle upon Tyne UK

**Keywords:** early‐onset arterial hypertension, focal segmental glomerulosclerosis, molecular diagnosis, nephronophthisis, *TTC21B*

## Abstract

Monogenic disorders of the kidney typically affect either the glomerular or tubulointerstitial compartment producing a distinct set of clinical phenotypes. Primary focal segmental glomerulosclerosis (FSGS), for instance, is characterized by glomerular scarring with proteinuria and hypertension while nephronophthisis (NPHP) is associated with interstitial fibrosis and tubular atrophy, salt wasting, and low‐ to normal blood pressure. For both diseases, an expanding number of non‐overlapping genes with roles in glomerular filtration or primary cilium homeostasis, respectively, have been identified. *TTC21B*, encoding IFT139, however has been associated with disorders of both the glomerular and tubulointerstitial compartment, and linked with defective podocyte cytoskeleton and ciliary transport, respectively. Starting from a case report of extreme early‐onset hypertension, proteinuria, and progressive kidney disease, as well as data from the Genomics England 100,000 Genomes Project, we illustrate here the difficulties in assigning this mixed phenotype to the correct genetic diagnosis. Careful literature review supports the notion that biallelic, often hypomorph, missense variants in *TTC21B* are commonly associated with early‐onset hypertension and histological features of both FSGS and NPHP. Increased clinical recognition of this mixed glomerular and tubulointerstitial disease with often mild or absent features of a typical ciliopathy as well as inclusion of *TTC21B* on gene panels for early‐onset arterial hypertension might shorten the diagnostic odyssey for patients affected by this rare tubuloglomerular kidney disease.

## 1 INTRODUCTION

Primary ciliopathies are a genetically heterogeneous group of disorders with mutations in genes whose encoded proteins contribute to the structure or function of the primary cilia or basal body (Braun & Hildebrandt, 2017). Primary cilia are hair‐like organelles that project from the cell surface with sensory and signaling functions (Reiter & Leroux, 2017). Typically, primary ciliopathies are multisystem disorders affecting organs that include the retina, brain, heart, skeletal system, liver and kidneys. The kidney phenotypes associated with primary ciliopathies are diverse and include enlarged polycystic kidneys such as seen in autosomal dominant and recessive polycystic kidney disease (ADPKD and ARPKD, respectively) and diseases that result in tubulointerstitial fibrosis and tubule basement membrane dysfunction such as nephronophthisis (NPHP) (Hildebrandt & Zhou, 2007). Numerous primary ciliopathy genes lead to NPHP‐like phenotypes and are collectively known as NPHP‐related ciliopathies (Braun & Hildebrandt, 2017; Devlin & Sayer, 2019). These typically present insidiously, often with bland urine, urinary concentration defects and progressive chronic kidney disease (CKD) leading to kidney failure (Srivastava, Molinari, Raman, & Sayer, 2017). Often the diagnosis is overlooked as features such as arterial hypertension (HTN) and proteinuria are absent and kidney ultrasound scanning may not reveal cysts or kidney enlargement, in contrast to ADPKD and ARPKD. In 20% of cases of NPHP, patients have extra‐renal phenotypes including retinal degeneration, liver fibrosis, and skeletal abnormalities, which often provide clues to an underlying primary ciliopathy.

In a similar way to NPHP‐related ciliopathies, caused by genes involved in ciliary function or structure, there are a growing number of monogenic causes of focal segmental glomerulosclerosis (FSGS), characterized by progressive scarring of the kidney glomeruli. FSGS is usually caused by genes encoding for proteins that contribute to the glomerular filtration barrier and podocyte function and there is typically no overlap between the molecular etiologies that cause FSGS and NPHP. In addition to these primary tubulointerstitial (NPHP) and glomerular (FSGS) disorders, there is however a rare hybrid form of “primary tubuloglomerular” disease that has been described in association with biallelic variants in *TTC21B* (Huynh Cong et al., 2014). *TTC21B* encodes for tetratricopeptide repeat domain‐containing protein 21B (or intraflagellar transport protein 139, IFT139) with an endogenous localization to the basal body and the ciliary axoneme in ciliated murine inner medullary collecting duct (mIMCD3) cells (Tran et al., 2008). In addition to this ciliary localization, IFT139 was also shown to distribute along the intracellular microtubule network in non‐ciliated adult podocytes (Huynh Cong et al., 2014). Biallelic mutations in *TTC21B* can cause both a distinct NPHP and FSGS phenotype, which presents with HTN, proteinuria, and progressive kidney failure (Bullich et al., 2017; Huynh Cong et al., 2014), reflecting this dual phenotype affecting both the glomerular and tubulointerstitial compartment (Huynh Cong et al., 2014).

Here we present two siblings who presented with extreme early‐onset HTN, proteinuria, and progressive CKD leading to kidney failure in whom we identified biallelic *TTC21B* variants as the underlying cause. We review their clinical features and describe their molecular genetic diagnosis. In addition, we review the genetic diagnoses associated with early‐onset hypertension in the Genomics England 100,000 Genomes Project and potential additional cases solved for *TTC21B* in this cohort. Finally, we perform an in‐depth literature review for the reported clinical spectrum of *TTC21B* mutations, with an emphasis on hypertensive phenotypes.

## 2 CASE REPORT

**FIGURE 1:**
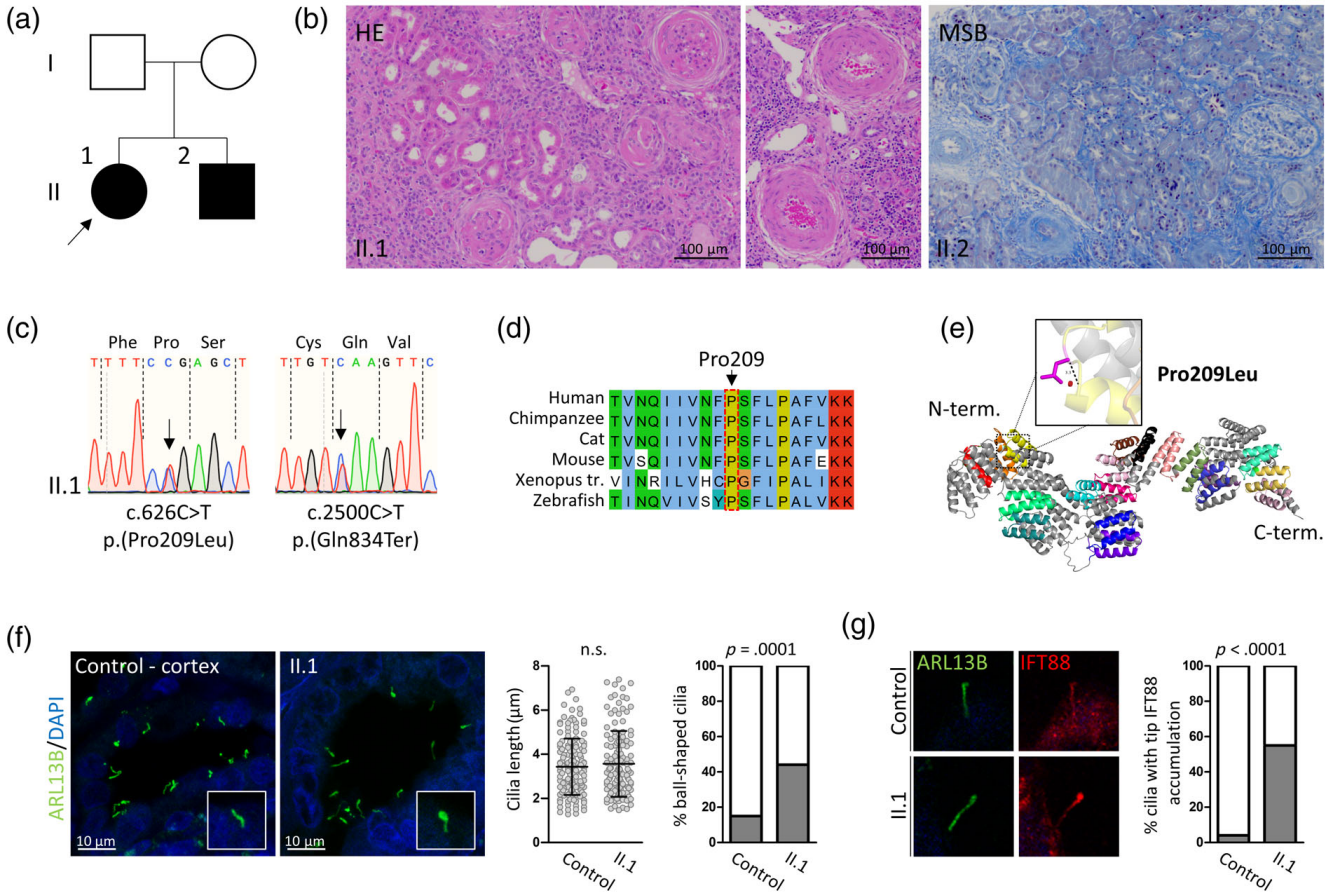
Biallelic pathogenic *TTC21B* variants in sibs with early‐onset severe arterial hypertension. (a) Pedigree of investigated family. Individuals with elevated arterial blood pressure and reduced kidney function are marked in black. Circles and squares denote females and males, respectively. (b) Histological analysis of nephrectomy specimen (for II.1) stained with hematoxylin and eosin (HE) and kidney biopsy specimen (for II.2) stained with Martius Scarlet Blue (MSB). For individual II.1, widespread global glomerulosclerosis with diffuse interstitial chronic inflammation and widespread tubular atrophy are noted. In addition, marked medial hypertrophy within interlobular arteries, reflecting systemic hypertension, is observed. For individual II.2, glomerulosclerosis with widespread periglomerular fibrosis and moderate chronic non‐specific tubulo‐interstitial damage are identified. Interlobular arteries show medial hypertrophy consistent with hypertension. (c) Sanger chromatograms confirming *TTC21B* variants in II.1. (d) Phylogenetic conservation of Pro209. Display generated using Clustal Omega Multiple Sequence Alignment (Sievers et al., 2011). (e) The predicted three‐dimensional structural model of human IFT139, encoded by *TTC21B* generated using AlphaFold Protein Structure Database (https://alphafold.ebi.ac.uk) and UniProtKB (https://www.uniprot.org/uniprot/) with associated code: Q7Z4L5. Alphafold sequence AF‐Q7Z4L5‐F1 was imported to PyMOL and labeled according to available UniProtKB data due to lack of crystal structure. PyMOL Mutation Wizard was used to model the most likely structural impact of p.(Pro209Leu). The protein's 19 tetratricopeptide (TPR) domains are color coded (red representing the first TPR domain to light pink representing the 19th). Missense SNV p.(Pro209Leu) is highlighted in pink with the most probable (72.4% likelihood) rotamer demonstrated. This change is occurring at a side loop within the distal end of TPR domain 3, one of the probable interaction scaffolds of this protein. The dashed black line demonstrates likely loss of the canonical geometry between 2 atoms in this loop with overlap of 3.3 Å. The red disk represents significant pairwise overlap of atomic van der Waals radii causing a likely structural “clash.” (f) Immunofluorescence staining for ciliary marker ARL13B (green) in patient II.1 nephrectomy sample and control human kidney cortex. The inset highlights a ball‐shaped cilia more frequently observed in patient tissue. Ciliary length is assessed based on ARL13B signal (ImageJ; Schneider, Rasband, & Eliceiri, 2012) using 131 (patient) and 148 (control) cilia on four and three different visual fields, respectively. n.s., not statistically significant (two‐tailed unpaired *t*‐test). The percentage of ball‐shaped cilia is assessed based on ARL13B signal and scoring 113 patient and 55 control cilia on eight and 10 different visual fields, respectively, from two independent experiments. *p*‐value calculated using Fisher's exact test. (g) Representative immunofluorescence staining for ciliary marker ARL13B (green) and IFT‐B component IFT88 (red) in patient II.1 nephrectomy sample and control human kidney cortex. Zoom‐ins on individual cilia. The percentage of cilia with tip IFT88 accumulation is assessed based on genotype‐blinded scoring of 113 patient and 55 control cilia on eight and 10 different visual fields, respectively, from two independent experiments. *p*‐value calculated using Fisher's exact test

The female proband (Figure 1a, II.1) presented at age 3 years and 6 months with pyrexia, cervical lymphadenopathy, and reduced appetite. On examination, she was pale and weighed 12.9 kg (ninth percentile) with a height of 88 cm (second percentile). Enlargement of cervical lymph nodes was confirmed, alongside hepatomegaly. She had an elevated systolic blood pressure (SBP) of 140 mmHg (>99th percentile). Urinalysis revealed proteinuria (albumin/creatinine ratio of 242 mg/mmol creatinine). Baseline investigations revealed raised serum creatinine (creatinine of 336 μmol/L, eGFR of 19 ml/min/1.73 m^2^, revised Schwartz equation), normal serum albumin (33 g/L), and severe anemia (Hb 6.6 g/dL). In addition, liver transaminases were elevated (ALP 531 IU/L, ALT 156 IU/L) (Table 1). Kidney ultrasound scan showed bilaterally small kidneys with increased echogenicity, as well as a loss of corticomedullary differentiation. Kidney biopsy showed sclerosed glomeruli, severe tubular atrophy, and severe tubulo‐interstitial fibrosis with interlobular arteries showing medial hypertrophy, consistent with the effects of hypertension (Figure 1b). Immunofluorescence staining was negative for complement and immunoglobulin deposition. The patient was managed with supportive care, including nutritional support, calcium, and vitamin D supplements. Her extreme hypertension was treated with Nifedipine, Atenolol, Hydralazine, and Ramipril. She was commenced on dialysis due to kidney failure (Stage 5 CKD) at age 3 years 7 months and as a result of persistent and poorly controlled HTN (SBP >140 mmHg despite four antihypertensives), a bilateral nephrectomy was performed. Following this, her SBP returned to the normal range with standard pharmacotherapy. This was subsequently followed by a deceased donor kidney transplant. Her liver function tests fluctuated in the upper‐normal or mildly elevated area but without progression of liver disease.

**TABLE 1:** Clinical information for affected sibs

Patient ID	Gender	Ethnicity	Age at referral	Clinical information
II.1	F	British	3 years, 6 months	Severe early‐onset hypertension (>99th percentile), proteinuria, kidney failure (3 years, 7 months), LV hypertrophy, liver function test abnormalities, growth retardation
II.2	M	British	1 year, 1 month	Severe early‐onset hypertension (>99th percentile), kidney failure (5 years)

Abbreviations: F, female; LV, left ventricle; M, male; m, months; y, years.

Her sibling, a 13‐month‐old boy (Figure 1a, II.2), presented just 3 days after the initial presentation of the proband with raised SBP of >100 mmHg (>99th percentile) and proteinuria (59 mg/mmol creatinine). Biochemistry showed reduced kidney function (eGFR of 56 ml/min/1.73 m^2^) and kidney ultrasound scan showed increased echogenicity, although the corticomedullary differentiation was preserved (Table 1). A kidney biopsy revealed sclerosed glomeruli and ischaemic glomeruli, moderate tubular atrophy, and chronic tubulointerstitial damage with interlobular arteries showing medial hypertrophy consistent with the effects of hypertension (Figure 1b). Immunofluorescence staining was negative for complement and immunoglobulin deposition. He was treated with the antihypertensives Atenolol, Furosemide, Nifedipine, and Spironolactone. His kidney function declined resulting in kidney failure by 5 years of age and was commenced on dialysis at this age. He received a deceased donor kidney transplant at age 6 years. His liver function tests were mildly elevated at last assessment.

Following informed consent, the patients II.1 and II.2 underwent genetic investigations. No parental DNA samples were available. According to the initial assumption that kidney disease was a consequence rather than cause of the early‐onset raised SBP, a monogenic cause of HTN was considered. *HSD11B2* gene sequencing did not reveal any underlying variants so the investigations were widened to whole‐genome sequencing (WGS) of the proband's (II.1) DNA in the context of the Genomics England 100,000 Genomes Project. An initial virtual panel for 26 and 61 high, intermediate, and low evidence genes for extreme early‐onset hypertension and kidney tubulopathies, respectively, was applied (Tables S1 and S2). This analysis revealed two heterozygous VUS (Table S3) but no clearly pathogenic changes. Following manual curation of the WGS data, biallelic rare variants in *TTC21B* were identified (Table 2), which were confirmed by Sanger sequencing in the proband as well as her affected brother (Figure 1c). The alleles included a heterozygous nonsense single‐nucleotide variant (SNV) p.(Gln834Ter) together with a heterozygous missense SNV p.(Pro209Leu), both of which were predicted to be pathogenic according to ACMG criteria (Richards et al., 2015) (Table 2). Homozygosity for the recurrent p.(Pro209Leu) variant has been previously described in relation with glomerular and tubulointerstitial damage characteristic of FSGS and NPHP, respectively (see discussion below) (Davis et al., 2011; Huynh Cong et al., 2014). Pro209 is phylogenetically conserved (Figure 1d) and *in silico* modeling based on alphaFold predictions suggest a likely structural “clash” at a side loop within the distal end of tetratricopeptide (TPR) domain 3 (Figure 1e). Functional studies have determined this variant to be hypomorphic, leading to reduced mutant protein expression and mislocalization (Davis et al., 2011; Huynh Cong et al., 2014). In animal models or cell studies, defects in IFT139 have been associated with ball‐shaped cilia due to the accumulation of IFT‐B components at the ciliary tip, consistent with the role of IFT139 in retrograde intraflagellar transport (Huynh Cong et al., 2014; Hirano, Katoh & Nakayama, 2017; Tran et al., 2008) but this has not previously been shown on patient tissues. Length of tubular epithelial cell cilia was not altered in proband (II.1) nephrectomy tissue when compared to control kidney cortex tissue (3.4 ± 1.3 μm vs. 3.6 ± 1.5 μm, *p* = .4, Figure 1f). We also assessed the percentage of ball‐shaped cilia as well as IFT‐B component IFT88 signal distribution in patient and control cilia. Genotype‐blinded scoring revealed a higher percentage of ball‐shaped cilia in proband (II.1) nephrectomy tissue (44.3% vs. 14.6% in controls, *p* = .0001) (Figure 1f) as well as a higher percentage of cilia with accumulation of IFT88 at the ciliary tip (54.9% vs. 3.6% in control tissue, *p* < .0001) (Figure 1g). Unfortunately, no material was available from patient II.2 to confirm these results. Nevertheless, these findings suggest indeed defective retrograde intraflagellar transport in patient cilia.

**TABLE 2:** Genetic variants in *TTC21B* detected in probands II.1 and II.2

Gene	Genomic coordinates (GRCh38)	Nucleotide change	Predicted amino acid change	gnomAD alleles^a^	ACMG classification
*TTC21B*	2:165907746:G:A	c.2500C>T	p.(Gln834Ter)	17/282292/0^b^	Pathogenic (PVS1, PM2, PP1, PP3, PP5)
*TTC21B*	2:165941111:G:A	c.626C>T	p.(Pro209Leu)	34/282510/0	Pathogenic (PS1, PS3, PM2, PP1, PP3, PP5)

*Note*: *TTC21B* transcript: NM_024753.5.

^a^ gnomAD alleles: allele count/ allele numbers/ number of homozygotes.

^b^ All 17 in Europeans.

Taken together, these clinical, genetic, and histological findings are consistent with pathogenic biallelic *TTC21B* variants leading to a mixed phenotype of interstitial changes typical of NPHP in association with features of extreme early‐onset HTN, proteinuria and glomerulosclerosis, both contributing to rapidly progressive kidney failure.

## 3 DISCUSSION AND REVIEW OF LITERATURE

*TTC21B* encodes IFT139, an essential component of the retrograde intraflagellar transport function of the primary cilium (Tran et al., 2008). Disruption of these IFT protein complexes has been known to cause ciliopathies, and pathogenic variants in *TTC21B* have been associated with multiple ciliopathy phenotypes consistent with this role in the cilium (Davis et al., 2011; McInerney‐Leo et al., 2015; Otto et al., 2011). In mice, homozygous null mutations in *Ttc21b* are embryonic lethal, with anatomical features of the fetus overlapping with some human ciliopathy phenotypes (Tran et al., 2008). Ciliopathy phenotypes associated with *TTC21B* variants include NPHP with or without extra‐renal features (Davis et al., 2011; Huynh Cong et al., 2014) and short‐rib dysplasia with or without polydactyly (Jeune asphyxiating thoracic dystrophy, JATD) (Davis et al., 2011; McInerney‐Leo et al., 2015). Intriguingly, biallelic variants in *TTC21B* may also cause a primary FSGS phenotype, which is more classically associated with podocytopathies and disorders of the glomerular filtration barrier rather than a primary ciliopathy (Bullich et al., 2017; Huynh Cong et al., 2014). Detailed histological examination of cases has revealed pathology in both the glomerular and tubulointerstitial compartment characteristic of FSGS and NPHP, respectively. This is also in line with the fact that IFT139 in human kidney is predominantly expressed in both glomeruli and distal tubules (Huynh Cong et al., 2014). A summary of the various human phenotypes linked to *TTC21B* variants can be found in Table 3 and Figure S1. The review was conducted by assessing studies that reported the phenotypes of patients with *TTC21B* variants. Published English‐language manuscripts were considered for review inclusion. A comprehensive search of PubMed using “(‘TTC21B’[Mesh] or ‘IFT139’[Mesh]) and (‘Kidney Disease’[Mesh] or ‘Renal Insufficiency, Chronic’[Mesh] or ‘FSGS’[tw] or ‘chronic kidney disease’[tw] or ‘tubulointerstitial disease’[tw])” was completed. The bibliographies of all included articles were examined to identify additional potentially relevant publications.

**TABLE 3:** Clinical phenotypes reported for biallelic *TTC21B* variants previously reported

Disease category/recruitment phenotype	Family	Origin	Patient ID	Sex	Age of presentation (years)	Kidney phenotype and associated features	Age at ESKD (years)	Other features	TTC21B mutation	Reference
NPHP	A3511	UK	21	n/a	n/a	NPHP	>8	HTN, chondrodysplasia, Bell's palsy	p.Arg411^a^ [Het] p.Thr483Aspfs^a^25 [Het]	Halbritter et al. (2013)
	A3260	USA	21	n/a	n/a	NPHP	3	Gastrointestinal tract malformation, situs inversus, polydactyly, polysplenia	p.Glu90^a^ [Hom]	
	A999	Germany	21	n/a	n/a	NPHP	2	Liver fibrosis	p.Pro209Leu [Het] p.Glu414^a^ [Het]	
	A4291	USA	21	n/a	n/a	NPHP	3	Liver fibrosis, cone‐shaped epiphysis (hands/ft)	p.Pro209Leu [Het] c.2868 + lG > T [Het]	
	A1065	Germany	21	n/a	n/a	NPHP	10	Situs inversus, hepatopathy	p.Pro209Leu [Het] p.Asp1308Gly [Het]	
Late‐onset FSGS	A	Tunisia	II‐2	F	15	Proteinuria n/a Biopsy not performed	15	HTN	p.Pro209Leu [Hom]	Huynh Cong et al. (2014)
			II‐3	F	23	Proteinuria FSGS lesions, stripes of tubulointerstitial fibrosis, atrophic tubules	32	HTN, myopia		
			II‐4	F	15	Proteinuria (<3.5 g/day) Biopsy not performed	27	HTN		
	B	Tunisia	II‐1	F	18	Proteinuria Biopsy not performed	27	HTN		
			II‐2	F	18	FSGS lesions, tubulointerstitial fibrosis	26	HTN, deafness, cerebral aneurysm		
			II‐3	M	9	Proteinuria (3 g/day) FSGS	16	HTN, cerebral aneurysm		
	C	Algeria	II‐1	M	22	Proteinuria (2.5 g/day) FSGS	22	HTN		
			II‐4	F	19	Proteinuria n/a FSGS lesions, stripes of tubulointerstitial fibrosis, atrophic tubules	23	HTN		
	D	Tunisia	II‐3	M	18	Proteinuria (2.9 g/day) FSGS lesions, tubulointerstitial fibrosis	27	HTN		
			II‐11	F	26	Proteinuria (7 g/day) MCNS lesions, foci of atrophic tubules, thickened TBM	35	HTN		
	E	Tunisia	II‐1	F	16	Proteinuria (<3.5 g/day) FSGS lesions	17	n/a		
	F	Algeria	II‐1	M	26	Proteinuria (1 g/day) FSGS lesions, severe tubulointerstitial lesions, thickened TBM	26	HTN		
	G	Algeria	II‐1	F	30	Proteinuria (2.2 g/day) FSGS lesions, tubulointerstitial fibrosis	34	Primary biliary cirrhosis		
NPHP	H	Portugal	II‐2	M	14	Proteinuria (<3.5 g/day) FSGS lesions, tubulointerstitial lesions, dedifferentiated tubules, thickened and multi‐layered TBM, atrophic tubules	20	HTN, severe scoliosis		
			II‐3	F	11	Proteinuria n/a Biopsy not performed	12	Bilateral hip osteotomy		
	I	Morocco	II‐1	F	10	Proteinuria (0.5 g/day) Global sclerosis lesions, severe tubulointerstitial fibrosis, atrophic tubules, thickened TBM, medullary cysts	14	HTN		
			II‐2	M	26	Proteinuria (1.5 g/day) Biopsy not performed	32	HTN		
	J	Portugal	II‐1	M	11	Proteinuria (0.25 g/day) FSGS lesions, tubulointerstitial fibrosis, foci of atrophic tubules, thickened TBM, medullar cysts	11	HTN, elevated liver enzymes		
FSGS	64	Spain	64‐1	F	4	Proteinuria (>3.5 g/day) FSGS lesions, interstitial inflammatory infiltrate	6	Myopia	p.Pro209Leu [Het] p.His426Asp [Het]	Bullich et al. (2017)
			64‐2	M	6	Proteinuria (>3.5 g/day) FSGS lesions, interstitial inflammatory infiltrate, atrophic tubules	8	Myopia	p.Pro209Leu [Het] p.His426Asp [Het]	
	22	Morocco	22	F	12	Proteinuria (>3.5 g/day) Global sclerosis lesions, tubulointerstitial fibrosis, atrophic tubules	14	HTN	p.Pro209Leu [Hom]	
	374	Morocco	374‐1	M	8	Proteinuria (>3.5 g/day) FSGS lesions, interstitial fibrosis, atrophic tubules	8	HTN, myopia	p.Pro209Leu [Hom]	
			374‐2	F	17	Proteinuria (>3.5 g/day) FSGS lesions, interstitial fibrosis, atrophic tubules	n/a	Myopia	p.Pro209Leu [Hom]	
NPHP		North Africa	7 Probands from 7 NPHP families	M (*n* = 5) F (*n* = 2)	25 ± 5	Proteinuria CKD stages 3–5 Severe vascular lesions, TMA, tubular lesions (*n* = 4)	29 ± 7	HTN emergency (*n* = 5), HTN retinopathy (*n* = 6), TMA (*n* = 2), multicystic kidney disease (*n* = 2), hepatic involvement (*n* = 5), LVH (*n* = 5)	p.Pro209Leu [Hom]	Doreille et al. (2021)
NPHP	F623	Portugal	II‐1	M	n/a	NPHP	n/a	n/a	p.Pro209Leu [Hom]	Davis et al. (2011)
			II‐2	F	n/a	NPHP	n/a	n/a	p.Pro209Leu [Hom]	
	A3214	Egypt	II‐1	M	n/a	NPHP	n/a	n/a	p.Pro209Leu [Hom]	
			II‐2	F	n/a	NPHP	n/a	n/a	p.Pro209Leu [Hom]	
	A34	Portugal	II‐1	F	Early‐onset^a^	NPHP	n/a	Extra‐renal features not specified	c.2758‐2A > G [Het] p.Pro209Leu [Het]	
	F244	Turkey	II‐5	F	Early‐onset^a^	NPHP	n/a	Extra‐renal features not specified	p.Cys552^a^ [Het] p.Pro209Leu [Het]	
	F514	N. Europe	II‐1	M	Early‐onset^a^	NPHP	n/a	Extra‐renal features not specified	p.Trp150Arg [Het] c.3264‐3C > G [Het]	
			II‐2	F	Early‐onset^a^	NPHP	n/a	Extra‐renal features not specified	p.Trp150Arg [Het] c.3264‐3C > G [Het]	
JATD	Fam3	N. Europe	B3	M	n/a	n/a	n/a	Thoracic dysplasia	p.Arg411^a^ [Het] p.Leu795Pro [Het]	
JATD	SKDP	UK	203.3	n/a	Adult	ESKD	3	Narrow thorax, brachydactyly, short long bones, scoliosis, hepatic cysts (adult)	c.152‐2A > G [Het] p.Leu1202Pro [Het]	McInerney‐Leo et al. (2015)
	SKDP	China	208.3	n/a	8	ESKD	8	Narrow thorax, polydactyly left hand and foot, brachydactyly, shortened long bones, short stature	p.Glu90_Ala91ins^a^ [Hom]	
NPHP	25	China	No. 25	M	7	Proteinuria (78 mg/kg/day) FSGS, tubulointerstitial lesions, dedifferentiated and atrophic tubules	8	HTN, situs inversus, short phalanges, physical retardation, neutropenia	c.2211 + 3A > G [Hom]	Zhang et al. (2018)
	34	China	No. 34	F	10 months	Proteinuria (158 mg/kg/day) FSGS glomerular lesions, tubulointerstitial lesions, dedifferentiated and atrophic tubules	1	HTN, hepatic fibrosis	p.Cys518Arg [Het] p.Arg486Lysfs^a^22 [Het]	
NPHP		China		F	3.5	(glomerular) proteinuria Renal dysfunction	4	HTN, situs inversus, short phalanges	p.Cys518Arg [Het] p.Met251Arg [Het]	Jian et al. (2019)
PKD, FSGS		Japan		M	Early infantile	Proteinuria (1–2 g/day) Multiple kidney cysts	15 months	Small liver cysts, dilated bile ducts	p.Tyr562Cys [Het] p.Ala857Thr [Het]	Hibino et al. (2020)
NPHP		Egypt	II‐1	M	8 months	Proteinuria (10.5 g/day) FSGS, severe chronic tubulointerstitial nephritis	1.5	HTN, right moderate hydronephrosis	p.Pro209Leu [Het] p.Trp150^a^ [Het]	Abo El Fotoh and Al‐Fiky (2020)
		Egypt	II‐2	M	n/a	ESKD	2.5	HTN	n/a	
FSGS		Ashkenazi‐Jewish		M	3	Proteinuria NA FSGS, C1q nephropathy, tubular atrophy, interstitial fibrosis and inflammation	4	HTN, biliary dysgenesis, myopia, PCD, elevated liver enzymes, mild portal fibrosis	c.1088‐1G > C [Het] p.Pro209Leu [Het]	Strong, Li, Mentch, and Hakonarson (2021)
JATD			Fam17	M	7	Renal insufficiency	n/a	Retrognathia, developmental delay	c.2758‐2A > G [Het] p.Ile1286Thr [Het]	Hammarsjö et al. (2021)
NPHP		Ukraine‐Jewish		M	10	Proteinuria Chronic glomerular sclerosis, mild/moderate tubular atrophy	6.5	Myopia, asymmetric rod‐cone dystrophy, pancreatitis, long palmar phalanges	p.Pro209Leu [Het] p.Glu509^a^ [Het]	Ben‐Yosef, Asia Batsir, Ali Nasser, and Ehrenberg (2021)
FSGS				F	6 months	Proteinuria (>3.5 g/day) FSGS, interstitial fibrosis, tubular atrophy	2.5	HTN, brachydactyly, skeletal abnormalities, cone‐shaped epiphyses	p.Pro209Leu [Het] p.Cys14Arg [Het]	Bezdíčka et al. (2021)
ESKD, biliary cirrhosis		North Africa		F	20	Proteinuria (1.5 g/day) Mild interstitial fibrosis and tubular atrophy	47	HTN, obesity, pre‐eclampsia, chronic cholestasis, liver fibrosis	p.Pro209Leu [Hom]	Gambino et al. (2021)

Abbreviations: CKD, chronic kidney disease; ESKD, end‐stage kidney disease; F, female; FSGS, focal segmental glomerulosclerosis; HTN, hypertension; JATD, Jeune asphyxiating thoracic dystrophy; LVH, left ventricular hypertrophy; M, male; MCNS, minimal change nephrotic syndrome; n/a, not available; NPHP, nephronophthisis; PKD, polycystic kidney disease; TBM, tubular basement membrane; TMA, thrombotic microangiopathy.

^a^ Age of onset was mentioned as early‐onset in the report.

Homozygous null alleles in *Ttc21b* are embryonic lethal in mice (Tran et al., 2008) and it is likely that biallelic truncating null variants are equally incompatible with survival in humans. For patients harboring other recessive *TTC21B* variants, a clear genotype phenotype correlation can be drawn. Patients harboring one truncating or splice site mutation in addition to a missense variant exhibit JATD or early‐onset NPHP with extra‐renal features, whereas patients carrying homozygous p.Pro209Leu variants display a primarily kidney phenotype with NPHP and often with glomerular involvement (FSGS). However, liver and skeletal involvement as well as high myopia have also been described in patients with biallelic missense variants, including homozygous p.Pro209Leu carriers (Bullich et al., 2017; Davis et al., 2011; Doreille et al., 2021). Our literature review indicated that overall, arterial HTN was the most prevalent extra‐renal manifestation reported in *TTC21B* cases. While arterial HTN was tendentially described more often in cases with the p.Pro209Leu variant (62.8% vs. 32.8% in non‐Pro209Leu cases, *p* = .12, Fisher's exact test), skeletal abnormalities, as seen in JATD, are significantly more common in non‐p.Pro209Leu patients (*p* = .0013, Fisher's exact test), in line with the suggested genotype phenotype correlations (Figure S1).

In addition to *TTC21B* being causative for recessive monogenic disease, rare pathogenic variants in *TTC21B* might also contribute to the mutational load in a diverse spectrum of ciliopathies and interact *in trans* with other disease‐causing genes. Thereby modifier alleles in *TTC21B* might potentially influence disease progression and phenotype expression, for instance promoting the development of kidney endophenotypes in ciliopathies (Davis et al., 2011). Similarly, a small study found that individuals with a heterozygous deleterious *TTC21B* variant in addition to cystic or glomerular disease‐causing mutations presented a more severe phenotype than expected (Bullich et al., 2017).

What is noteworthy in the two cases we present is the extreme early‐onset HTN that was associated with their presentation, which initially side‐tracked the molecular genetic investigations to determining a monogenic cause for this. Indeed, early‐onset arterial HTN is usually not observed in NPHP‐related ciliopathies. Reviewing the published *TTC21B* cases looking for reports of extreme elevated arterial blood pressure is interesting. The initial *TTC21B* case reports were associated with ciliopathy phenotypes that included NPHP as well as JATD (Davis et al., 2011). However precise details concerning each clinical presentation, such as proteinuria and SBP readings, were not provided. The first reference of elevated blood pressure with *TTC21B* variants was in an 8‐year‐old child with NPHP. The child had compound heterozygous nonsense (p.Arg411Ter) and frameshifting (p.Thr483Aspfs*25) variants in the *TTC21B* gene, presenting with chondrodysplasia, Bell's palsy, and HTN alongside the typical NPHP features (Halbritter et al., 2013). The concept of *TTC21B* variants being causative of elevated SBP was broached later on, in a study reporting seven out of 18 ciliopathy patients with extreme, early‐onset SBP, and end‐organ damage (Doreille et al., 2021). Six out of these 7 patients had homozygous *TTC21B* p.Pro209Leu missense variants. As several patients developed HTN prior to kidney dysfunction, the authors proposed the pathophysiology of hypertension as endothelial ciliary dysfunction, secondary to the defectively encoded IFT39 protein. The authors also coined the term “nephroangionophthisis” to represent the NPHP phenotypic spectrum encompassing systemic HTN and vascular injury. The mean age of onset of HTN in that study was 23 years, that is, much later than the cases that we describe here. Furthermore, the majority of patients reported by Doreille et al. had liver function test abnormalities, in line with the observed elevated liver transaminases in our index case (Doreille et al., 2021).

Further substantial evidence was provided by Huynh Cong et al. in a study linking biallelic p.Pro209Leu variants of *TTC21B* with familial FSGS phenotypes that presented with proteinuria and HTN. Of the 10 families reported in the paper, eight pedigrees had elevated SBP (15 out of 18 patients, 83%). It was also reported in this study that the p.Pro209Leu mutation arose from a founder effect, originating from individuals of North African or Portuguese descent (Huynh Cong et al., 2014). On detailed examination, the authors confirmed that homozygous carriers of the p.Pro209Leu variant had both histological hallmarks of FSGS and NPHP (tubular basement membrane thickening) (Huynh Cong et al., 2014). Elegant studies in mature unciliated human podocytes revealed that the p.Pro209Leu variant leads to actin cytoskeleton alterations, such as short and disorganized stress fibers and microtubule rearrangement into bike wheel–like shape. These findings suggest that the FSGS phenotype associated with certain *TTC21B* variants is not due to ciliary defects but rather reflects the role of IFT139 in the regulation of podocyte cytoskeleton architecture (Huynh Cong et al., 2014).

Following this study, other cases of *TTC21B* mutations and elevated SBP have been reported throughout literature (Table 3), despite it not being the main phenotype of investigation. In total, 56% percent of cases with *TTC21B* pathogenic variants and extrarenal findings reported in literature were reported to have HTN (Figure S1). The cases with HTN usually present in late childhood or early adulthood. Proteinuria is one of the major findings on presentation and kidney biopsy revealed mostly FSGS lesions in these cases with HTN (Table 3). It is interesting to note that blood pressure returned on target levels after nephrectomy in our index case as well as in other reported cases after kidney transplantation, supporting the notion that IFT139 defects in the kidney are primarily responsible for the elevated blood pressure (Gambino et al., 2021).

**FIGURE 2:**
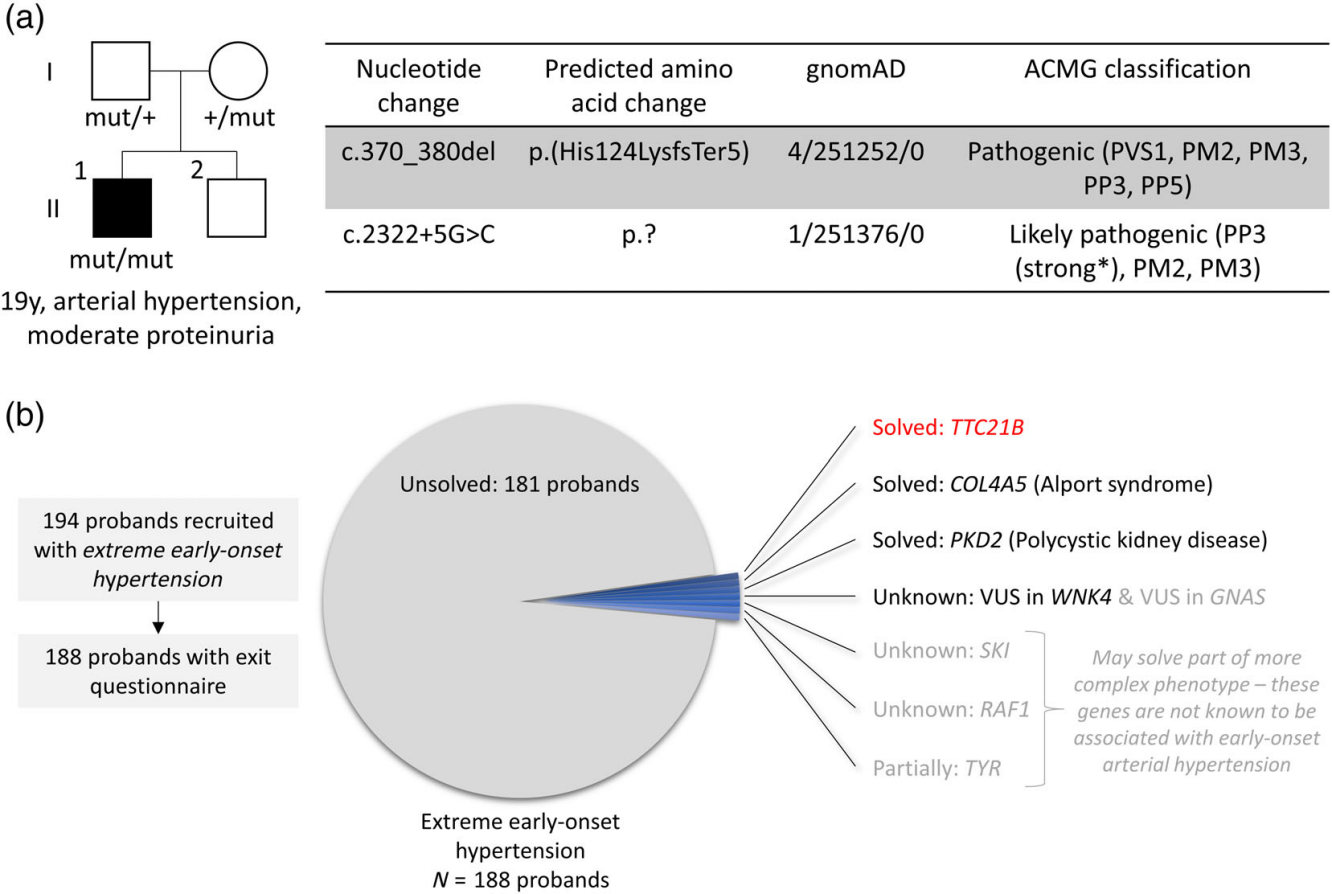
Genomics England 100,000 Genomes Project analysis for *TTC21B* and early‐onset hypertension. (a) Pedigree of additional family identified in the 100,000 Genomes dataset with proband II.1 carrying compound heterozygous *TTC21B* variants *in trans*. The affected individual is marked in black with phenotype description below. Information on identified *TTC21B* variants is provided in table. *TTC21B* transcript: NM_024753.5; gnomAD data (Karczewski et al., 2020) presented as allele count, total allele numbers, number of homozygotes; *In silico analysis for splicing effect: SpliceAI delta score for splice donor loss: 0.55—MaxEntScan alt: 6.1, ref: 11.0, diff: 4.9. (b) Analysis of genetic diagnoses for 100,000 Genomes Project rare disease patients recruited under “Extreme early‐onset hypertension”. Out of 188 probands, only seven probands are definitively, potentially or partially solved (including our index family with biallelic *TTC21B* variants). In four probands, variants in genes that explain their arterial HTN have been detected (red or black). In three other probands, variants have been reported that may explain part of their more complex phenotype but that are not known to be associated with systemic HTN (gray). Only variants in primary kidney disease genes (*TTC21B, COL4A5*, *PKD2*, and *WNK4*) have been detected as a cause for arterial HTN

Examination of the Genomics England 100,000 Genomes rare disease dataset revealed an additional patient with biallelic predicted pathogenic *TTC21B* variants *in trans*. The first variant is a frameshifting variant while the second variant is a rare intronic SNV predicted to lead to splice donor loss (Figure 2a). Of interest, this 19‐year‐old patient has been recruited with proteinuric renal disease and is also affected with arterial HTN. We also examined genetic diagnoses of Genomics England 100,000 Genomes rare disease patients recruited under the disease group “extreme early‐onset hypertension”. Among the 188 probands for which a Genomics England report is available (“exit questionnaire”), only seven are potentially, partially, or definitively solved after WGS (including our initial case that was solved after manual curation of WGS data) (Figure 2b). Among those, only four probands are solved for genes that explain their systemic HTN while in three other probands, variants have been reported that may explain part of their more complex phenotype but that are not known to be associated with systemic HTN. Interestingly, the few solved cases with systemic HTN were all caused by primary kidney disease gene variants (*TTC21B*, *COL4A5, PKD2*, and *WNK4*), of which only *WNK4* (Pseudohypoaldosteronism, type IIB, MIM #614491) is currently listed on the extreme early‐onset HTN panel from Genomics England. Of note, the two probands solved for *PKD2* and *COL4A5* presented with typical disease features (kidney and liver cysts, microscopic haematuria and ocular manifestations, respectively), so that the appropriate kidney panels have been run alongside the early‐onset arterial HTN panel. These exploratory data highlight the low yield of the virtual gene panel for systemic arterial HTN. There is obvious overlap between genes involved in arterial HTN and genes implicated in other disorders, most prominently kidney disease. To increase the rate of molecular diagnoses, it appears nevertheless reasonable to add genes to the extreme early‐onset HTN panel for which arterial HTN can be an early or leading clinical manifestation (Figure 2b).

**FIGURE 3:**
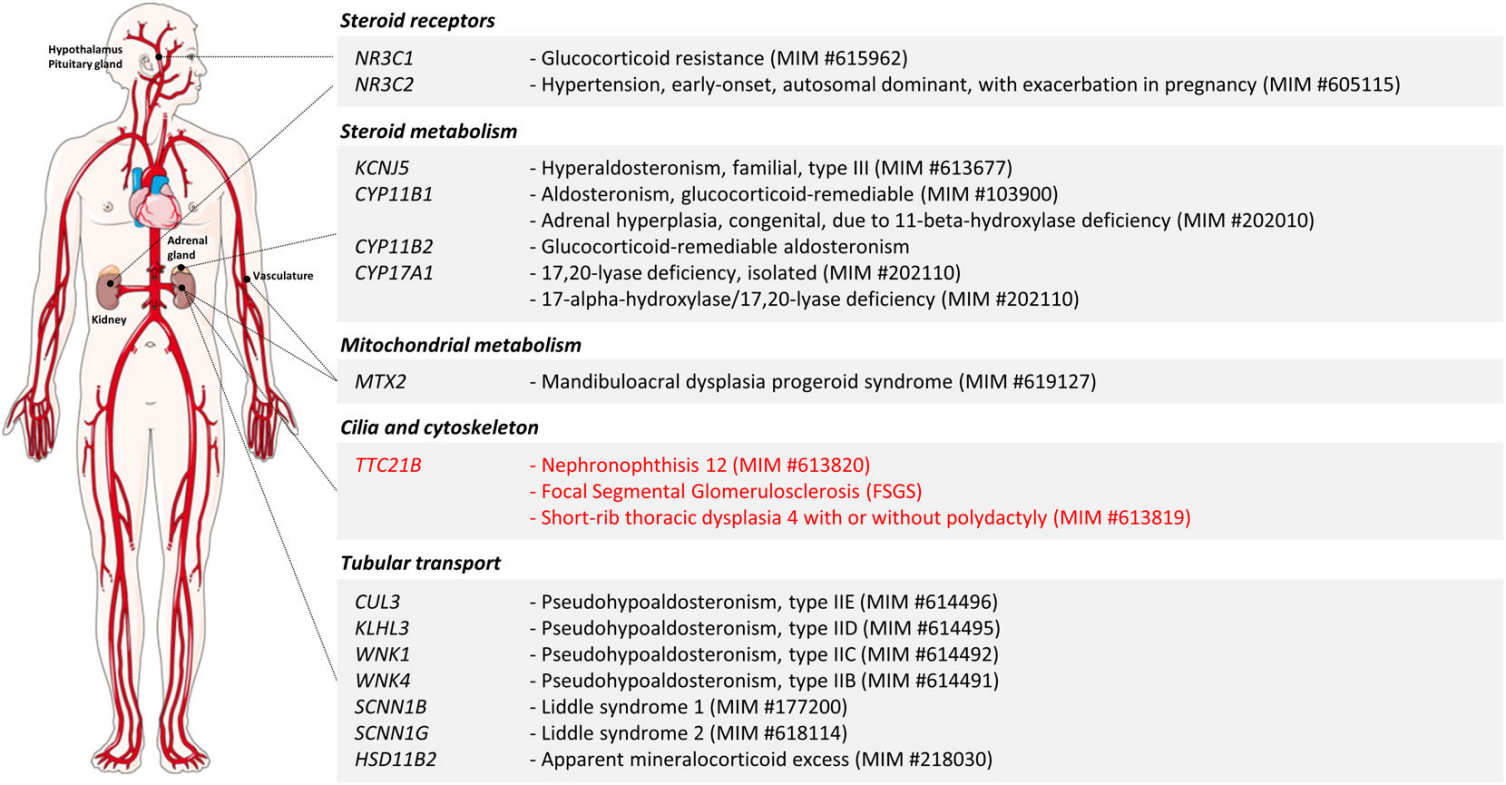
High evidence etiologies for monogenic arterial hypertension included in the Genomics England PanelApp extreme early‐onset hypertension virtual panel (Version 1.14). Genetic etiologies are classified according to their main function and site of action relating to blood pressure regulation. *TTC21B* is not currently included in the extreme early‐onset hypertension virtual panel (Version 1.14) (https://panelapp.genomicsengland.co.uk/panels/314/) and would be the first representative of a new class of HTN‐associated genes with roles in the primary cilium and cytoskeleton. Figure generated using Servier Medical Art (https://smart.servier.com/)

Despite HTN being a recurring theme in biallelic *TTC21B* variants, this gene is not currently recognized as a causative gene for extreme early‐onset HTN in Genomics England PanelApp virtual panels (Table S1). As we mentioned, WGS and an applied virtual panel of HTN genes did not currently include *TTC21B*, resulting in diagnostic delay. Based on the two cases we present and the overwhelming evidence in the published literature, it seems reasonable to propose the addition of *TTC21B* to the extreme early‐onset HTN gene panels to ensure a timelier genetic diagnosis. Currently, there are 14 high evidence genes included in the Genomics England PanelApp virtual panel for extreme early‐onset HTN, with well‐defined roles in kidney tubular transport, adrenal gland steroid metabolism, steroid receptors, and mitochondrial metabolism. *TTC21B* would be the first representative of a new class of HTN‐associated genes with roles in the primary cilium and cytoskeleton (Figure 3 and Table S1).

## CONFLICTS OF INTEREST

The authors declare no conflicts of interest.

## ETHICS STATEMENT

This study was approved by the North East—Newcastle & North Tyneside 1 Research Ethics Committee (18/NE/350). All included individuals provided informed and written consent.

## Supporting information


**Appendix** **S1:** Supporting Information.

## Data Availability

The data that support the findings of this study are available on request from the corresponding author. The data are not publicly available due to privacy or ethical restrictions.

## Genomics England Research Consortium

27th May 2021

John C. Ambrose^1^; Prabhu Arumugam^1^; Roel Bevers^1^; Marta Bleda^1^; Freya Boardman‐Pretty^1,2^; Christopher R. Boustred^1^; Helen Brittain^1^; Mark J. Caulfield^1,2^; Georgia C. Chan^1^; Greg Elgar^1,2^; Tom Fowler^1^; Adam Giess^1^; Angela Hamblin^1^; Shirley Henderson^1,2^; Tim J. P. Hubbard^1^; Rob Jackson^1^; Louise J. Jones^1,2^; Dalia Kasperaviciute^1,2^; Melis Kayikci^1^; Athanasios Kousathanas^1^; Lea Lahnstein^1^; Sarah E. A. Leigh^1^; Ivonne U. S. Leong^1^; Javier F. Lopez^1^; Fiona Maleady‐Crowe^1^; Meriel McEntagart^1^; Federico Minneci^1^; Loukas Moutsianas^1,2^; Michael Mueller^1,2^; Nirupa Murugaesu^1^; Anna C. Need^1,2^; Peter O'Donovan^1^; Chris A. Odhams^1^; Christine Patch^1,2^; Mariana Buongermino Pereira^1^; Daniel Perez‐Gil^1^; John Pullinger^1^; Tahrima Rahim^1^; Augusto Rendon^1^; Tim Rogers^1^; Kevin Savage^1^; Kushmita Sawant^1^; Richard H. Scott^1^; Afshan Siddiq^1^; Alexander Sieghart^1^; Samuel C. Smith^1^; Alona Sosinsky^1,2^; Alexander Stuckey^1^; Mélanie Tanguy^1^; Ana Lisa Taylor Tavares^1^; Ellen R. A. Thomas^1,2^; Simon R. Thompson^1^; Arianna Tucci^1,2^; Matthew J. Welland1; Eleanor Williams^1^; Katarzyna Witkowska^1,2^; Suzanne M. Wood^1,2^.

^1^ Genomics England, London, UK.

^2^ William Harvey Research Institute, Queen Mary University of London, London, EC1M 6BQ, UK.
