# Supplementary material for: Biallelic variants in TTC21B as a rare cause of early‐onset arterial hypertension and tubuloglomerular kidney disease
Source: Am J Med Genet C Semin Med Genet. 2022 Mar 15;190(1):109–20. doi: 10.1002/ajmg.c.31964 (PMC9314882; doi:10.1002/ajmg.c.31964)
Supplement: Supplementary file 1 — Appendix S1: Supporting Information. [file AJMG-190-109-s001.docx]

Supplementary Information

Biallelic variants in *TTC21B* as a rare cause of early-onset arterial hypertension and tubuloglomerular kidney disease

Supplementary Figure 1

Supplementary Tables 1-3

**Figure S1: Prevalence of extra-renal phenotypes in reported *TTC21B* cases.**

**
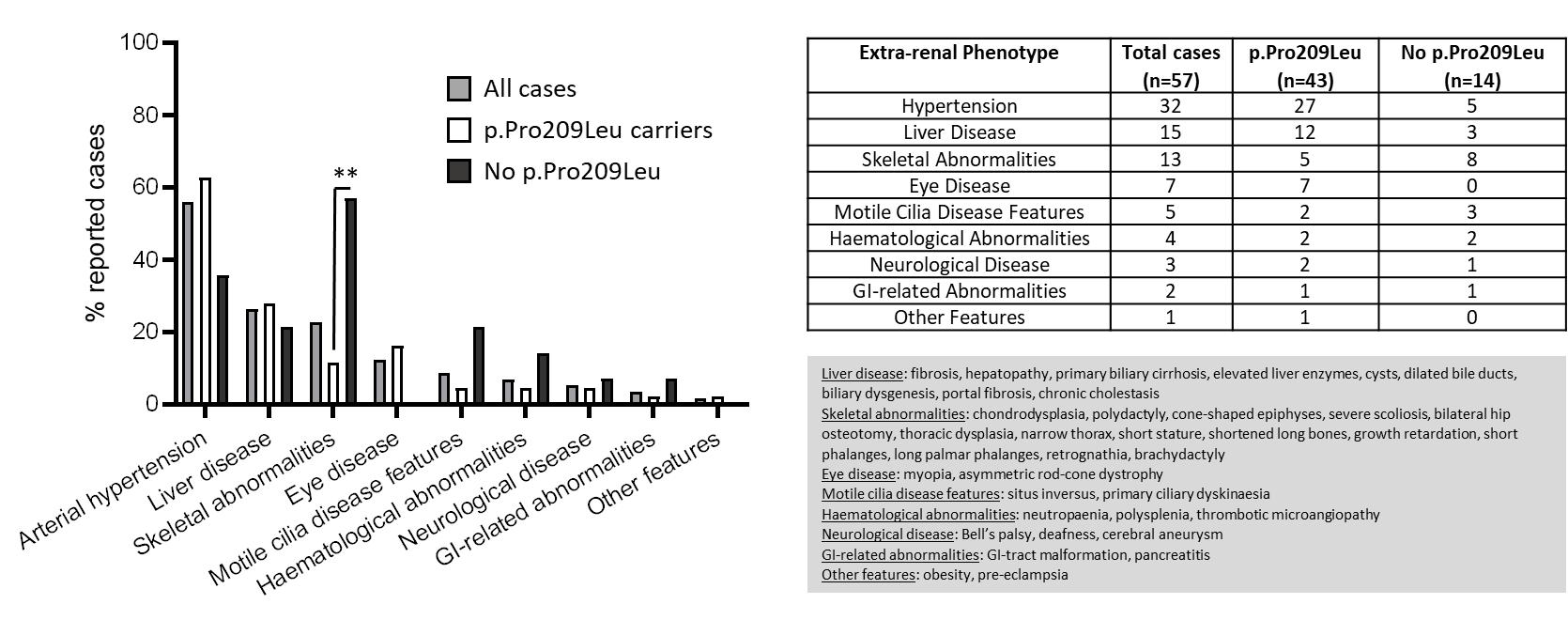
**

Prevalence (%) of extra-renal manifestations in all *TTC21B* (n=57), p.Pro209Leu heterozygotes and homozygotes (n=43) and cases without p.Pro209Leu (n=14) reported in literature. Arterial hypertension is the most prevalent extra-renal phenotype and while arterial hypertension is tendentially more prevalent in cases with the p.Pro209Leu variant (62.8% vs. 32.8%, p=0.12, Fisher’s exact test), skeletal abnormalities are significantly more common in non-p.Pro209Leu patients (p=0.0013, Fisher’s exact test). The phenotypic details contributed to the prevalence groups are specified in the grey box.

**Table S1: Genomics England 100,000 Genomes Project extreme early-onset hypertension PanelApp (Version 1.14) rare disease virtual panel including 26 high evidence (green), intermediate evidence (yellow) and low evidence (red) genes.**

| Gene Symbol | Sources (; separated) | Model_Of_Inheritance | Phenotypes, Omim, Orphanet, HPO | Ensembl Id (GRch38) |
| --- | --- | --- | --- | --- |
| *CUL3* | Expert;Expert Review Green;Illumina TruGenome Clinical Sequencing Services;Literature;Radboud University Medical Center, Nijmegen;UKGTN | MONOALLELIC, autosomal or pseudoautosomal, NOT imprinted | Pseudohypoaldosteronism, type IIE, 614496 | ENSG00000036257 |
| *CYP11B1* | Expert;Expert Review Green;Illumina TruGenome Clinical Sequencing Services;Radboud University Medical Center, Nijmegen;UKGTN | MONOALLELIC, autosomal or pseudoautosomal, NOT imprinted | Adrenal hyperplasia, congenital, due to 11-beta-hydroxylase deficiency, 202010;Aldosteronism, glucocorticoid-remediable, 103900;Early onset hypertension with raised urinary 18-hydroxy-steroids; steroid-sensitive. | ENSG00000160882 |
| *CYP11B2* | Expert Review Green;Radboud University Medical Center, Nijmegen | MONOALLELIC, autosomal or pseudoautosomal, NOT imprinted | Aldosterone to renin ratio raised, Glucucorticoid-remediable hyperaldosteronism | ENSG00000179142 |
| *CYP17A1* | Emory Genetics Laboratory;Expert;Expert Review Green;Illumina TruGenome Clinical Sequencing Services;Radboud University Medical Center, Nijmegen;UKGTN | BIALLELIC, autosomal or pseudoautosomal | 17,20-lyase deficiency, isolated 202110;17-alpha-hydroxylase/17,20-lyase deficiency 202110 | ENSG00000148795 |
| *HSD11B2* | Expert;Expert Review Green;Literature;Radboud University Medical Center, Nijmegen;UKGTN | BIALLELIC, autosomal or pseudoautosomal | Apparent mineralocorticoid excess, 218030 | ENSG00000176387 |
| *KCNJ5* | Emory Genetics Laboratory;Expert;Expert Review Green;Illumina TruGenome Clinical Sequencing Services;Radboud University Medical Center, Nijmegen | MONOALLELIC, autosomal or pseudoautosomal, NOT imprinted | Hyperaldosteronism, familial, type III 613677 | ENSG00000120457 |
| *KLHL3* | Expert list;Expert Review Green;Illumina TruGenome Clinical Sequencing Services;Literature;Radboud University Medical Center, Nijmegen;UKGTN | BOTH monoallelic and biallelic, autosomal or pseudoautosomal | Pseudohypoaldosteronism, type IID, 614495 | ENSG00000146021 |
| *MTX2* | Expert Review;Expert Review Green;Literature | BIALLELIC, autosomal or pseudoautosomal | Mandibuloacral dysplasia;lipodystrophy;arterial calcification;severe hypertension | ENSG00000128654 |
| *NR3C1* | Expert;Expert Review;Expert Review Green;Illumina TruGenome Clinical Sequencing Services;Radboud University Medical Center, Nijmegen | MONOALLELIC, autosomal or pseudoautosomal, NOT imprinted | Glucocorticoid resistance 615962 | ENSG00000113580 |
| *NR3C2* | Expert list;Expert Review Green;Literature | MONOALLELIC, autosomal or pseudoautosomal, NOT imprinted | Hypertension, early-onset, autosomal dominant, with exacerbation in pregnancy, 605115 | ENSG00000151623 |
| *SCNN1B* | Expert;Expert Review Green;Illumina TruGenome Clinical Sequencing Services;Radboud University Medical Center, Nijmegen;UKGTN | BOTH monoallelic and biallelic, autosomal or pseudoautosomal | Bronchiectasis with or without elevated sweat chloride 1 211400;Liddle syndrome, 177200;Pseudohypoaldosteronism, type I, 264350 | ENSG00000168447 |
| *SCNN1G* | Expert;Expert Review Green;Illumina TruGenome Clinical Sequencing Services;Radboud University Medical Center, Nijmegen;UKGTN | BOTH monoallelic and biallelic, autosomal or pseudoautosomal | Bronchiectasis with or without elevated sweat chloride 3, 613071;Liddle syndrome, 177200;Pseudohypoaldosteronism, type I, 264350 | ENSG00000166828 |
| *WNK1* | Expert;Expert Review Green;Illumina TruGenome Clinical Sequencing Services;Radboud University Medical Center, Nijmegen;UKGTN | BOTH monoallelic and biallelic, autosomal or pseudoautosomal | Neuropathy, hereditary sensory and autonomic, type II, 201300; Pseudohypoaldosteronism, type IIC, 614492 | ENSG00000060237 |
| *WNK4* | Expert list;Expert Review Green;Illumina TruGenome Clinical Sequencing Services;Radboud University Medical Center, Nijmegen;UKGTN | MONOALLELIC, autosomal or pseudoautosomal, NOT imprinted | Pseudohypoaldosteronism, type IIB, 614491 | ENSG00000126562 |
| *CACNA1H* | Expert Review Amber;Literature | MONOALLELIC, autosomal or pseudoautosomal, imprinted status unknown | Hyperaldosteronism, familial, type IV 617027;{Epilepsy, childhood absence, susceptibility to, 6} 611942;{Epilepsy, idiopathic generalized, susceptibility to, 6} 611942 | ENSG00000196557 |
| *AGT* | Expert Review Red;Radboud University Medical Center, Nijmegen;UKGTN | Unknown | {Hypertension, essential, susceptibility to}, 145500;{Preeclampsia, susceptibility to}Renal tubular dysgenesis, 267430 | ENSG00000135744 |
| *AGTR1* | Expert Review Red;Radboud University Medical Center, Nijmegen | BIALLELIC, autosomal or pseudoautosomal | Hypertension, essential, 145500;Renal tubular dysgenesis, 267430 | ENSG00000144891 |
| *BMPR2* | Radboud University Medical Center, Nijmegen | MONOALLELIC, autosomal or pseudoautosomal, NOT imprinted | Pulmonary hypertension, familial primary, 1, with or without HHT, 178600, Pulmonary hypertension, primary, fenfluramine or dexfenfluramine-associated, 178600, Pulmonary venoocclusive disease 1, 265450 | ENSG00000204217 |
| *CAV1* | Expert Review Red;Radboud University Medical Center, Nijmegen | MONOALLELIC, autosomal or pseudoautosomal, NOT imprinted | Lipodystrophy, congenital generalized, type 3, 612526;Pulmonary hypertension, primary, 3, 615343 | ENSG00000105974 |
| *CPS1* | Expert Review Red;Radboud University Medical Center, Nijmegen | BIALLELIC, autosomal or pseudoautosomal | Carbamoylphosphate synthetase I deficiency, 237300;{Pulmonary hypertension, neonatal, susceptibility to}, 615371;{Venoocclusive disease after bone marrow transplantation} | ENSG00000021826 |
| *CYP21A2* | Expert;Expert Review Red | BIALLELIC, autosomal or pseudoautosomal | Adrenal hyperplasia, congenital, due to 21-hydroxylase deficiency 201910;Hyperandrogenism, nonclassic type, due to 21-hydroxylase deficiency 201910 | ENSG00000231852 |
| *KCNK3* | Expert Review Red;Radboud University Medical Center, Nijmegen | MONOALLELIC, autosomal or pseudoautosomal, NOT imprinted | Pulmonary hypertension, primary, 4, 615344 | ENSG00000171303 |
| *PNMT* | Expert Review Red;Radboud University Medical Center, Nijmegen | Unknown | NA | ENSG00000141744 |
| *PTGIS* | Expert Review Red;Radboud University Medical Center, Nijmegen | Other - please specifiy in evaluation comments | Hypertension, essential, 145500 | ENSG00000124212 |
| *SARS2* | Expert Review Red;Illumina TruGenome Clinical Sequencing Services;Radboud University Medical Center, Nijmegen;UKGTN | BIALLELIC, autosomal or pseudoautosomal | Hyperuricemia, pulmonary hypertension, renal failure, and alkalosis, 613845 | ENSG00000104835 |
| *SMAD9* | Expert Review Red;Radboud University Medical Center, Nijmegen | MONOALLELIC, autosomal or pseudoautosomal, NOT imprinted | Pulmonary hypertension, primary, 2 615342 | ENSG00000120693 |

**Table S2: Genomics England 100,000 Genomes Project renal tubulopathies PanelApp (Version 2.30) rare disease virtual panel including 61 high evidence (green), intermediate evidence (yellow) and low evidence (red) genes.**

| Gene Symbol | Sources(; separated) | Model_Of_Inheritance | Phenotypes, Omim, Orphanet, HPO | Ensembl Id (GRch38) |
| --- | --- | --- | --- | --- |
| AP2S1 | Expert Review Green;NHS GMS | MONOALLELIC, autosomal or pseudoautosomal, NOT imprinted | Familial hypocalciuric hypercalcemia type III 600740 | ENSG00000042753 |
| AQP2 | Expert Review Green;NHS GMS;Other | BOTH monoallelic and biallelic, autosomal or pseudoautosomal | Diabetes insipidus, nephrogenic, 125800;Nephrogenic diabetes insipidus | ENSG00000167580 |
| ATP1A1 | Expert Review Green;NHS GMS | MONOALLELIC, autosomal or pseudoautosomal, NOT imprinted | Hypomagnesemia, seizures, and mental retardation 2 618314;Charcot-Marie-Tooth disease, axonal, type 2DD, 618036 | ENSG00000163399 |
| ATP6V0A4 | Eligibility statement prior genetic testing;Expert;Expert Review Green;Illumina TruGenome Clinical Sequencing Services;NHS GMS;Radboud University Medical Center, Nijmegen;UKGTN | BIALLELIC, autosomal or pseudoautosomal | Distal renal tubular acidosis 3, with or without sensorineural hearing loss, OMIM:602722 | ENSG00000105929 |
| ATP6V1B1 | Eligibility statement prior genetic testing;Expert;Expert Review Green;Illumina TruGenome Clinical Sequencing Services;NHS GMS;Radboud University Medical Center, Nijmegen;UKGTN | BIALLELIC, autosomal or pseudoautosomal | Renal tubular acidosis with deafness, 267300;Distal Renal Tubular Acidosis with Progressive Nerve Deafness;Distal renal tubular acidosis | ENSG00000116039 |
| AVPR2 | Expert Review Green;NHS GMS;Other | X-LINKED: hemizygous mutation in males, monoallelic mutations in females may cause disease (may be less severe, later onset than males) | Diabetes insipidus, nephrogenic, OMIM:304800;Nephrogenic syndrome of inappropriate antidiuresis, OMIM:300539 | ENSG00000126895 |
| BSND | Eligibility statement prior genetic testing;Expert Review Green;NHS GMS | BIALLELIC, autosomal or pseudoautosomal | Hypokalaemic alkalosis with hypercalciuria;Bartter syndrome type 4a;Sensorineural deafness with mild renal dysfunction MIM 602522 | ENSG00000162399 |
| CA2 | Expert;Expert Review Green;Illumina TruGenome Clinical Sequencing Services;NHS GMS;Radboud University Medical Center, Nijmegen;UKGTN | BIALLELIC, autosomal or pseudoautosomal | Osteopetrosis, autosomal recessive 3, with renal tubular acidosis, 259730;Osteopetrosis with Renal Tubular Acidosis | ENSG00000104267 |
| CASR | Expert Review Green;NHS GMS | BOTH monoallelic and biallelic, autosomal or pseudoautosomal | Hypocalcemia, autosomal dominant, (with Bartter syndrome), 601198;Hypocalciuric hypercalcemia, type I, 145980;Hyperparathyroidism, neonatal, 239200 | ENSG00000036828 |
| CLCNKB | Eligibility statement prior genetic testing;Expert Review Green;NHS GMS | BIALLELIC, autosomal or pseudoautosomal | Hypokalaemic alkalosis with hypomagnesaemia & hypocalciuria;Bartter syndrome, type 3, 607394 | ENSG00000184908 |
| CLDN16 | Expert Review Green;NHS GMS | BIALLELIC, autosomal or pseudoautosomal | Hypomagnesemia 3, renal 248250 | ENSG00000113946 |
| CLDN19 | Expert Review Green;NHS GMS | BIALLELIC, autosomal or pseudoautosomal | Hypomagnesemia 5, renal, with ocular involvement, 248190 | ENSG00000164007 |
| CTNS | Emory Genetics Laboratory;Expert Review;Illumina TruGenome Clinical Sequencing Services;Literature;NHS GMS;Radboud University Medical Center, Nijmegen;UKGTN | BIALLELIC, autosomal or pseudoautosomal | Cystinosis, atypical nephropathic 219800;Cystinosis, late-onset juvenile or adolescent nephropathic 219900;Cystinosis, nephropathic 219800;Cystinosis, ocular nonnephropathic 219750 | ENSG00000040531 |
| CUL3 | Expert Review Green;NHS GMS | MONOALLELIC, autosomal or pseudoautosomal, NOT imprinted | Pseudohypoaldosteronism, type IIE, 214496 | ENSG00000036257 |
| CYP24A1 | Expert Review Green;NHS GMS | BIALLELIC, autosomal or pseudoautosomal | Hypercalcemia, infantile, 1 143880 | ENSG00000019186 |
| FAH | Emory Genetics Laboratory;Expert Review;Illumina TruGenome Clinical Sequencing Services;Literature;Radboud University Medical Center, Nijmegen;UKGTN | BIALLELIC, autosomal or pseudoautosomal | Tyrosinemia, type I 276700 | ENSG00000103876 |
| GATM | Expert Review Green;NHS GMS | MONOALLELIC, autosomal or pseudoautosomal, NOT imprinted | Renal fanconi syndrome and kidney failure (no MIM number);Cerebral creatine deficiency syndrome 3, 612718 (AR) | ENSG00000171766 |
| GNA11 | Expert Review Green;NHS GMS | MONOALLELIC, autosomal or pseudoautosomal, NOT imprinted | Hypocalcemia, autosomal dominant 2 615361 | ENSG00000088256 |
| HNF1B | Expert Review Green;NHS GMS | MONOALLELIC, autosomal or pseudoautosomal, NOT imprinted | Renal cysts and diabetes syndrome, 137920;Diabetes mellitus, noninsulin-dependent, 125853 | ENSG00000275410 |
| KCNJ1 | Eligibility statement prior genetic testing;Expert Review Green;NHS GMS | BIALLELIC, autosomal or pseudoautosomal | Hypokalaemic alkalosis with hypercalciuria;Type 2 Bartter syndrome;often initial transient hyperkalemia;Bartter syndrome, type 2, 241200 | ENSG00000151704 |
| KCNJ10 | Expert Review Green;NHS GMS | BIALLELIC, autosomal or pseudoautosomal | SESAME/EAST syndrome, 612780 | ENSG00000177807 |
| KLHL3 | Expert Review Green;NHS GMS | BOTH monoallelic and biallelic, autosomal or pseudoautosomal | Pseudohypoaldosteronism, type IID, 614495 | ENSG00000146021 |
| MAGED2 | Expert Review Green;NHS GMS | X-LINKED: hemizygous mutation in males, biallelic mutations in females | Bartter syndrome, type 5, antenatal, transient, 300971 | ENSG00000102316 |
| NR3C2 | Expert Review Green;NHS GMS | MONOALLELIC, autosomal or pseudoautosomal, NOT imprinted | Pseudohypoaldosteronism type I, autosomal dominant, 177735;Hypertension, early-onset, autosomal dominant, with exacerbation in pregnancy, 605115 no inheritance pattern | ENSG00000151623 |
| REN | Expert Review Green;NHS GMS | BOTH monoallelic and biallelic, autosomal or pseudoautosomal | Hyperuricemic nephropathy, familial juvenile 2, 613092;Renal tubular dysgenesis 267430 AR | ENSG00000143839 |
| SCNN1A | Expert Review Green;NHS GMS | BIALLELIC, autosomal or pseudoautosomal | Pseudohypoaldosteronism, type I, 264350;?Liddle syndrom 3, 618126;Bronchiectasis with or without elevated sweat chloride 2 613021 | ENSG00000111319 |
| SCNN1B | Eligibility statement prior genetic testing;Expert Review Green;NHS GMS | BOTH monoallelic and biallelic, autosomal or pseudoautosomal | Pseudohypoaldosteronism, type I, 264350 | ENSG00000168447 |
| SCNN1G | Expert Review Green;NHS GMS | BOTH monoallelic and biallelic, autosomal or pseudoautosomal | Pseudohypoaldosteronism, type I, 264350 | ENSG00000166828 |
| SLC12A1 | Eligibility statement prior genetic testing;Expert Review Green;NHS GMS | BIALLELIC, autosomal or pseudoautosomal | Type 1 Bartter syndrome: infantile onset, pregnancy noted for polyhydramnios. Hyperprostagladinuria. Hypokalaemia and metabolic alkalosis +/- nephrocalcinosis;Bartter syndrome, type 1, 601678 | ENSG00000074803 |
| SLC12A3 | Eligibility statement prior genetic testing;Expert Review Green;NHS GMS | BIALLELIC, autosomal or pseudoautosomal | Hypokalaemic alkalosis with hypomagnesaemia & hypocalciuria;Gitelman syndrome, 263800 | ENSG00000070915 |
| SLC22A12 | Expert Review Green;NHS GMS | BIALLELIC, autosomal or pseudoautosomal | Hypouricemia, renal, 220150 | ENSG00000197891 |
| SLC2A9 | Expert Review Green;NHS GMS | BOTH monoallelic and biallelic, autosomal or pseudoautosomal | Hypouricemia, renal, 2, 612076;{Uric acid concentration, serum, QTL 2}, 612076 | ENSG00000109667 |
| SLC4A1 | Eligibility statement prior genetic testing;Expert;Expert Review Green;Illumina TruGenome Clinical Sequencing Services;NHS GMS;Radboud University Medical Center, Nijmegen;UKGTN | BOTH monoallelic and biallelic, autosomal or pseudoautosomal | Distal Renal Tubular Acidosis, Dominant;Ovalocytosis;Distal renal tubular acidosis;Renal tubular acidosis, distal, AD,179800;Renal tubular acidosis, distal, AR, 611590;Cryohydrocytosis, 185020;Ovalocystois, SA type 166900;Spherocytoisis type 4, 612653;various blood group associations. | ENSG00000004939 |
| SLC4A4 | Expert;Expert Review Green;Illumina TruGenome Clinical Sequencing Services;NHS GMS;Radboud University Medical Center, Nijmegen | BIALLELIC, autosomal or pseudoautosomal | Renal tubular acidosis, proximal, with ocular abnormalities, 604278;Proximal Renal Tubular Acidosis with Ocular Abnormalities;Proximal Renal Tubular Acidosis with Ocular Abnormalities (recessive). | ENSG00000080493 |
| SLC5A2 | Expert Review Green;NHS GMS | BOTH monoallelic and biallelic, autosomal or pseudoautosomal | Renal glucosuria, 233100 | ENSG00000140675 |
| TRPM6 | Expert list;Expert Review Green;NHS GMS | BIALLELIC, autosomal or pseudoautosomal | Hypomagnesemia 1, intestinal, 602014 | ENSG00000119121 |
| UMOD | Expert Review Green;NHS GMS | MONOALLELIC, autosomal or pseudoautosomal, NOT imprinted | Hyperuricemic nephropathy, familial juvenile 1, 162000;Glomerulocystic kidney disease with hyperuricemia and isosthenuria, 609886;Medullary cystic kidney disease 2, 603860 | ENSG00000169344 |
| WNK4 | Expert Review Green;NHS GMS | MONOALLELIC, autosomal or pseudoautosomal, NOT imprinted | Pseudohypoaldosteronism, type IIB, 614491 | ENSG00000126562 |
| CLCNKA | Expert Review Amber;NHS GMS | BIALLELIC, autosomal or pseudoautosomal | Bartter syndrome, type 4b, digenic 613090 | ENSG00000186510 |
| CLDN10 | Expert Review Amber;NHS GMS | BIALLELIC, autosomal or pseudoautosomal | Hypokalemic-alkalotic salt-losing tubulopathy (no OMIM number);HELIX syndrome, 617671 | ENSG00000134873 |
| EHHADH | Expert;Expert Review Amber;NHS GMS | MONOALLELIC, autosomal or pseudoautosomal, NOT imprinted | ?Fanconi renotubular syndrome 3, OMIM:615605;L-bifunctional protein deficiency;Metabolic acidosis;Increased amino acids in urine | ENSG00000113790 |
| FOXI1 | Expert Review;Expert Review Amber;Literature;NHS GMS | BIALLELIC, autosomal or pseudoautosomal | deafness;renal tubular acidosis;Early onset sensorinerual deafness and distal renal tubular acidosis (no OMIM number);Enlarged vestibular aqueducts, 6007910 | ENSG00000168269 |
| FXYD2 | Expert Review Amber;NHS GMS | MONOALLELIC, autosomal or pseudoautosomal, NOT imprinted | Hypomagnesemia 2, renal, 154020 | ENSG00000137731 |
| GNAS | Expert Review Amber;NHS GMS | MONOALLELIC, autosomal or pseudoautosomal, paternally imprinted (maternal allele expressed) | Unexplained hyponatremia in infancy, severe early-onset gonadotrophin-independent precocious puberty and skeletal abnormalities. | ENSG00000087460 |
| HNF4A | Expert list;Expert Review Amber | MONOALLELIC, autosomal or pseudoautosomal, NOT imprinted | Fanconi renotubular syndrome 4, with maturity-onset diabetes of the young, MIM#616026 | ENSG00000101076 |
| SARS2 | Expert Review Amber;Literature | BIALLELIC, autosomal or pseudoautosomal | Hyperuricemia, pulmonary hypertension, renal failure, and alkalosis 613845;Progressive Spastic Paresis | ENSG00000104835 |
| SLC2A2 | Expert list;Expert Review Amber | BIALLELIC, autosomal or pseudoautosomal | Fanconi-Bickel syndrome, MIM# 227810 | ENSG00000163581 |
| VIPAS39 | Expert Review;Expert Review Amber | BIALLELIC, autosomal or pseudoautosomal | Arthrogryposis, renal dysfunction, and cholestasis 2 #613404 | ENSG00000151445 |
| VPS33B | Expert Review Amber;Literature | BIALLELIC, autosomal or pseudoautosomal | Arthrogryposis, renal dysfunction, and cholestasis 1 #208085 | ENSG00000184056 |
| WDR72 | Expert Review Amber;Literature | BIALLELIC, autosomal or pseudoautosomal | distal RTA;hereditary distal renal tubular acidosis | ENSG00000166415 |
| WNK1 | Expert Review Amber;NHS GMS | MONOALLELIC, autosomal or pseudoautosomal, NOT imprinted | Pseudohypoaldosteronism, type IIC, 614492 | ENSG00000060237 |
| ABCG2 | NHS GMS | Unknown | Serum uric acid concentration and susceptibility to gout, 138900 | ENSG00000118777 |
| CLCN5 | Expert Review Red;NHS GMS | X-LINKED: hemizygous mutation in males, monoallelic mutations in females may cause disease (may be less severe, later onset than males) | Dent disease, 300009. Hypophosphatemic rickets, 300554. Nephrolithiasis, type I, 310468. Proteinuria, low molecular weight, with hypercalciuric nephrocalcinosis, 308990 | ENSG00000171365 |
| EGF | NHS GMS |  | Hypomagnesemia 4, renal, 611718 | ENSG00000138798 |
| KCNA1 | NHS GMS |  | Autosomal dominant hypomagnesemia;Episodic ataxia/myokymia syndrome,160120 | ENSG00000111262 |
| OCRL | Expert Review Red;NHS GMS | X-LINKED: hemizygous mutation in males, biallelic mutations in females | Dent disease 2, 300555. Lowe syndrome, 309000 | ENSG00000122126 |
| SLC34A1 | Expert Review Red;NHS GMS | BOTH monoallelic and biallelic, autosomal or pseudoautosomal | Hypercalcemia, infantile, 2, MIM 616963;Nephrolithiasis/osteoporosis, hypophosphatemic, 1, 612286;?Fanconi renotubular syndrome 2 613388 | ENSG00000131183 |
| SLC34A3 | Expert Review Red;NHS GMS | BIALLELIC, autosomal or pseudoautosomal | Hypophosphatemic rickets with hypercalciuria, 241530 | ENSG00000198569 |
| SLC9A3R1 | Expert Review Red;NHS GMS | MONOALLELIC, autosomal or pseudoautosomal, NOT imprinted | Nephrolithiasis/osteoporosis, hypophosphatemic, 2, 612287 | ENSG00000109062 |
| XPR1 | Expert Review;Expert Review Red;Literature | Unknown | Fanconi syndrome;hypophosphatamia | ENSG00000143324 |

**Table S3: Variants of unknown significance detected in proband II.1 after whole genome sequencing and application of virtual panels (Tables S2-3)**

| Gene | Genomic coordinates (GRCh38) | Nucleotide change | Predicted amino acid change | gnomAD alleles | ACMG classification |
| --- | --- | --- | --- | --- | --- |
| *SLC5A2* | 16:31487674:G:A | c.800G>A (het) | p.(Arg267Gln) (het) | 4/250070/0 | VUS (PM2, PP3) |
| *WNK4* | 17:42785358:C:G | c.1352C>G (het) | p.(Pro451Arg) (het) | 3/233850/0 | VUS (PM2, BP4) |

*SLC5A2* transcript: NM_003041.3; *WNK4* transcript: NM_032387.4; Het: heterozygote
